# Impairment in social cognition in people with substance use disorders

**DOI:** 10.3389/fpsyt.2025.1574483

**Published:** 2025-05-27

**Authors:** Adolfo Piñón-Blanco, Sonia Rodrigues, Joana Teixeira, Catia Coutinho, Isabela Faria, Ilda Murta, Ana Isabel Tavares, Luis Iglesias-Rejas, Indalecio Carrera-Machado, Alejandro Garcia-Caballero, Olga Gutiérrez-Martínez, Francisco Otero-Lamas, Carlos Spuch

**Affiliations:** ^1^ Drug Dependency Assistance Unit of the City, Council of Vigo, CEDRO, Vigo, Spain; ^2^ RD24/0003/0024 Group, Red de investigación de atención primaria en adicciones (RIAPAD), Barcelona, Spain; ^3^ Translational Neuroscience Group, Galicia Sur Health Research Institute (IIS Galicia Sur), SERGAS-UVIGO, University of Vigo, Vigo, Spain; ^4^ Integrated Response Center, CRI of Porto Ocidental, Institute for Addictive Behaviours and Dependencies, I.P. (ICAD.IP), Porto, Portugal; ^5^ ULS de São José, Centro Clínico Académico de Lisboa, Faculdade de Medicina da Universidade de Lisboa, Unidade de Alcoologia do Hospital Júlio de Matos, Lisboa, Portugal; ^6^ Unidade Local de Saúde de Coimbra, Coimbra, Portugal; ^7^ Research Department, Citizens’ Association for the Fight Against Drugs (ACLAD), A Coruña, Spain; ^8^ Schizophrenia Unit, Ourense University Hospital Complex Day Hospital, Ourense, Spain; ^9^ Psychiatric Service, Vigo University Hospital Complex, Vigo, Spain; ^10^ GCV17/SAM1 Group, CIBERSAM, Madrid, Spain

**Keywords:** substance use disorders, social cognition, emotional recognition, empathy, theory of mind, attributional style

## Abstract

**Introduction:**

Substance use disorders are associated with impairments in various neuropsychological functions. We evaluated potential alterations in social cognition and differences between men and women in individuals with substance use disorders undergoing treatment at Addiction and Dependency Intervention Centers in Portugal.

**Methods:**

The assessment utilized the Ekman 60 Faces Test (EFT), Interpersonal Reactivity Index (IRI), Hinting Task, and Ambiguous Intentions Hostility Questionnaire (AIHQ).

**Results:**

Results showed that 70.2% of participants exhibited social cognition impairments (50% of women and 79.5% of men). Compared to non-clinical populations, individuals with social cognition impairments displayed significant differences in recognizing emotions such as happiness, fear, sadness, disgust, anger, and in the total EFT score. Differences were also observed in the fantasy and personal distress dimensions of the IRI, as well as in hostility, intentionality, and aggression biases on the AIHQ. Slight differences were found between men and women, but were not statistically significant.

**Discussion:**

We discuss the clinical relevance of social cognition alterations and their potential utility in improving diagnostic and therapeutic processes for individuals with substance use disorders.

## Introduction

Substance Use Disorders (SUD) are an increasing challenge for healthcare systems worldwide, driven by, among other factors, the high number of individuals using drugs within a context of expansive production, market growth, and accessibility. It is estimated that over 290 million people used drugs in 2022, a 20% increase compared to the previous decade. Approximately 64 million individuals suffer from SUD, yet only 1 in 11 people receive treatment, 1 in 18 among women compared to 1 in 7 among men (1). Furthermore, the multifactorial etiopathogenesis of SUD complicates the development of improved preventive and therapeutic strategies. Despite advances in understanding the biological, psychological, and social mechanisms underlying the disorders (2, 3), the translation of this knowledge into clinical practice remains limited.

From a neurobiological and neuropsychological perspective, one widely studied area of interest is the impairment of cognitive and executive functions in individuals with SUD, including their cause-effect relationships, role in the disease progression, and impact on treatment efficacy. It is well-established that neurocognitive alterations associated with drug use involve both prefrontal and hippocampal cognitive domains. A substantial body of evidence consistently links these impairments to premature treatment dropout, clinical outcomes, and relapse rates (4–9).

Additionally, research on enhancing cognitive functions as an adjunct strategy in treating individuals with SUS has shown positive results. These improvements are observed not only in cognitive variables but also in several clinical parameters related to treatment effectiveness (10–12).

In this context, the study of social cognition (SC) has recently gained prominence. SC is a complex, multifactorial construct referring to the ability to build representations of relationships between oneself and others and to use these representations flexibly to guide social behavior (13). It encompasses the set of cognitive processes used to decode and encode the social world (14) or that are activated in social interaction contexts (15). The components of SC remain a topic of debate, but from a clinical perspective, five subdomains have been proposed as particularly relevant: theory of mind (ToM), social perception, social knowledge, attributional biases, and emotional processing (16).

While most clinical research in this field has primarily focused on schizophrenia and autism spectrum disorders, the inclusion of SC as a primary domain in the RDoC framework (17) and its designation as one of the six domains of cognitive function in the DSM-5 (18) have renewed and expanded interest in studying SC across mental disorders. Additionally, it is known that male and female brains exhibit neurofunctional differences in various aspects of SC, such as face processing, facial expression recognition, response to infant schema, the ability to perceive faces in objects, processing of social interactions, empathy for others’ pain, interest in social information, processing of gestures and actions, biological motion, and erotic and affective stimuli (19). Thus, it is essential to deepen our understanding of gender differences in SC.

Most studies on SC in patients with SUD have been conducted in the context of alcohol (20, 21) and stimulants (22–24). However, there is also evidence related to cannabis (25, 26), opioids (27), steroids (28), and polydrug use (29–32), as well as, on the bidirectional relationship between SC and the risk of developing SUD, especially in youth populations (33). Although scientific literature consistently highlights the presence of SC impairments in individuals with SUD and their potential clinical relevance (34, 35), the diagnostic and therapeutic procedures commonly employed for treating these patients often do not incorporate specific resources to address SC.

The main objective of this study was to assess the presence of impairments in SC, including emotion recognition, empathy, theory of mind, and attributional style, in a clinical sample of individuals with SUD undergoing treatment, and to investigate potential gender differences.

Based on this, the following hypotheses were formulated:

Primary hypothesis: Most individuals with SUD will exhibit clinical impairment in at least one subdomain of social cognition, with particular emphasis on emotion recognition and empathy.

Secondary hypothesis: Males with SUD will show greater deficits in social cognition than females, particularly in facial emotion recognition and empathic responses.

Exploratory hypothesis: Individuals with impaired SC will have significantly different scores in attributional biases and theory of mind compared to normative values from the non-clinical population.

## Materials and methods

### Study design

This was a multicenter, cross-sectional, prospective study with neuropsychological measures. It was conducted between June and December 2022 at Addiction and Dependency Intervention Centers in Portugal: Integrated Response Center of Western Porto, Therapeutic Team of Matosinhos, University Hospital Center of Coimbra, and Lisbon Psychiatric Hospital Center. Clinical trial: NCT06363331.

### Participants

A total of 57 individuals were recruited from the Addiction and Dependency Intervention Centers in Portugal based on the following criteria:

*Inclusion Criteria:*

- Diagnosis of SUD according to the DSM-5 (36).
- Age 18 or older.
- Capacity to consent (competence).
- Ability to read and write.
- Read the project’s information sheet and sign the informed consent form.

*Exclusion Criteria:*

- Diagnosis of intellectual disability (IQ < 70).
- Moderate or severe neurological damage.
- Presence of an acute psychiatric condition.
- Abstinence period of fewer than 15 days.

### Participant selection

Participants were selected through convenience sampling, using a consecutive sampling method of patients undergoing treatment as they were admitted to these care facilities.

During a follow-up therapy session, participants were informed about the study characteristics and voluntarily agreed to participate by signing the informed consent form. This research, approved by the ARS Norte Research Ethics Committee, guaranteed: The participant’s right to withdraw from the study at any time without penalty and complete confidentiality of all collected data.

### Sample size determination

The sample size calculation was based on standard statistical parameters for comparative studies between two independent groups. Considering a statistical power of 80% (1 - β = 0.80), a significance level of 5% (α = 0.05, two-tailed), and a large effect size (Cohen’s d = 0.8), the minimum required sample size would be 52 participants (26 per group), according to estimates generated by G*Power software (version 3.1).

Therefore, the final sample of 57 participants was deemed sufficient to detect large effects with adequate statistical power. However, to detect medium (d = 0.5) or small (d = 0.2) effects, approximately 128 and 394 participants would be required, respectively, which exceeds the sample size obtained in this study.

### Instruments

A sociodemographic data collection questionnaire and a battery of neuropsychological tests were administered. **Table 1** lists the tests used and the SC subdomains evaluated.

**Table 1:** Instruments used for assessing social cognition subdomains.

TEST	SUBDOMAINS OF SOCIAL COGNITION
**Ekman 60 Faces Test (EFT)**	Social cognition: emotional recognition subdomain
**The Interpersonal Reactivity Index (IRI)**	Social cognition: empathy subdomain
**Hinting Task**	Social cognition: theory of mind subdomain
**Ambiguous Intentions Hostility Questionnaire (AIHQ)**	Social cognition: attributional style subdomain

EFT, Ekman 60 Faces Test; IRI, Interpersonal Reactivity Index; AIHQ, Ambiguous Intentions Hostility Questionnaire. Each instrument assesses one or more subdomains of social cognition, as specified below.

*Ekman 60 Faces Test (EFT)* (37): This test includes 60 photographs of faces displaying expressions of six basic emotions: anger, disgust, sadness, fear, surprise, and happiness. A general score of 60 indicates the best possible performance, with each basic emotion having a maximum score of 10 points.

*The Interpersonal Reactivity Index* (IRI) (38, 39): A Likert scale that evaluates four dimensions of empathy:

- Fantasy (F): The tendency to identify with fictional characters.
- Perspective Taking (PT): The ability to adopt others’ points of view.
- Empathic Concern (EC): Sympathy and concern for others’ suffering.
- Personal Distress (PD): Feelings of discomfort when witnessing others in distress.

*Hinting Task* (40): This test evaluates the ability to infer the true intention behind hints expressed in ten short stories involving two characters. Participants are asked what the character in the story meant to convey. The ability to infer the underlying, true intention of these indirect language uses involves employing Theory of Mind (ToM).

*Ambiguous Intentions Hostility Questionnaire* (AIHQ) (41, 42): This test assesses attributional biases through various vignettes that describe situations where the intentions of characters are ambiguous, intentional, or accidental. Participants are asked to rate on a Likert scale:

- AIHQ-HB: Hostility bias—why they think the protagonist acted this way.
- AIHQ-IS: Intentionality bias—whether the action was deliberate.
- AIHQ-BS: Blame bias—how much they blame the protagonist.
- AIHQ-AS: Anger bias—how angry the situation makes them feel.
- AIHQ-AB: Aggressiveness bias—how they would respond to the situation.

Higher scores reflect more hostile, negative, personal, and aggressive attributions.

### Procedure

Sociodemographic and clinical variables were obtained from the center’s database. Neuropsychological tests were administered according to the application and scoring guidelines in their respective manuals. Testing was conducted over two 45-minute sessions under similar conditions.

All study participants have a DSM-5 diagnosis of SUD (Substance Use Disorder) confirmed by addiction specialists from the Portuguese ICAD (Intervention in Addictive Behaviors and Dependencies). The diagnostic assessments were conducted at specialized Addiction Intervention and Dependency Treatment Centers in Portugal (Porto, Coimbra, and Lisbon).

All recruited participants underwent clinical assessment and initial SC screening through emotional recognition and empathy evaluations. SC impairment was determined using the following criteria:

### Inclusion criteria for social cognition impairment

The impairment criterion required either:

- A score below 42 on emotional recognition (EFT), and/or
- Scores more than 1 standard deviation below the mean in any of the four empathy dimensions (Fantasy, Perspective-Taking, Empathic Concern, or Personal Distress).

### Assessment methodology

The SC impairment criteria used for the EFT (37) and IRI (39) were based on scoring ranges derived from healthy populations. For the IRI specifically, we used normative data from the Portuguese adaptation of the test.

Participants identified as having SC impairment according to screening criteria also underwent evaluations of ToM and attributional style subdomains.

### Administration protocol

Trained psychologists conducted all test administrations and scoring procedures following standardized instructions validated for the Portuguese population.

**Figure 1:**
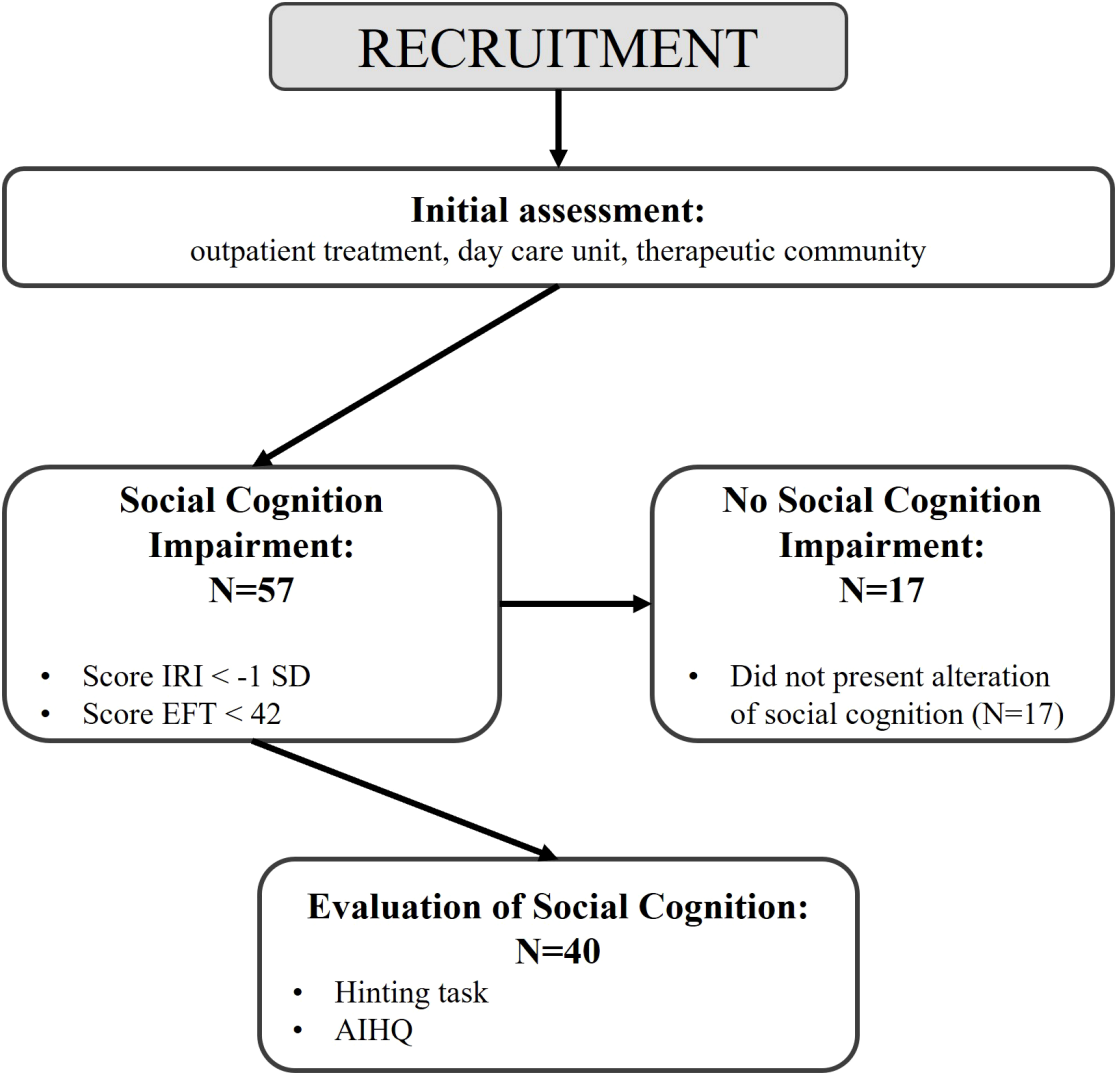
Flowchart of protocol.

**Figure 1** illustrates the evaluation procedure performed at each study phase and the participants involved in each phase.

### Data analysis

A descriptive analysis was performed for all variables. For qualitative variables, absolute frequencies and their respective percentages were calculated. For quantitative variables, measures of central tendency (mean and standard deviation) were computed.

For variables with n<30, the Mann-Whitney U test was used to analyze potential differences between means of NSCI and SCI variables across each dimension of the EFT and IRI tests within female and male groups.

When the sample size exceeded 30, a one-sample t-test was applied to continuous variables (EFT, IRI, AIHQ, and HINTING TASK scores) to examine differences between normative values and obtained mean scores for each test.

### Statistical analyses

An independent samples Student’s t-test was used to analyze potential significant differences between mean scores measured in z-scores by sex. For group comparisons (e.g., impaired vs. unimpaired SC; males vs. females), Mann-Whitney U tests and independent samples t-tests were used according to variable distribution, with a significance level set at 5% (*p* < 0.05). Comparisons between clinical sample scores and normative values from the non-clinical population were conducted using one-sample t-tests.

All analyses were conducted using IBM SPSS Statistics (version 29), with a significance level set at 0.05.

## Results

We recruited 57 patients for the study; of all of them, 17 (29.8%) did not show SC impairment according to the initial screening criteria, while 40 (70.2%) exhibited some level of impairment. Regarding sex, a total of 18 women were recruited, of whom 50% (n=9) showed SC impairment. Among the 39 men, 79.5% (n=31) presented SC impairment. **Table 2** displays the descriptive analysis of sociodemographic and clinical variables.

**Table 2:** Sociodemographic and clinical features: Total Sample and Social Cognition Impairment (SCI) vs. No Impairment (NSCI) groups.

Demography	Total population	No Impairment Group	Social Cognition Impairment
	Total N=57 (100%)	NSCI N=17 (29,8%)	SCI N=40 (70,2%)
**Age (years)** ^a^	47,23 ± 10,53	46,18 ± 7,85	47,68 ± 11,54
**Sex** ^b^ • Male • Female	39 (68,4%) 18 (31,6%)	8 (47,1%) 9 (52,9%)	31 (77,5%) 9 (22,5%)
**Educational level** ^b^ • Primary education • Secondary education • Others/Jobb training • University education	24 (42,1%) 18 (31,6%) 4 (7,0%) 11 (19,3%)	5 (29,4%) 5 (29,4%) 2 (11,8%) 5 (19,4%)	19 (47,5%) 13 (32,5%) 2 (5,0%) 5 (15,0%)
**Main drug of current consumption** ^b^ • Cocaine • Cannabis • Alcohol • Opiates	8 (14,0%) 4 (7,0%) 35 (61,4%) 10 (17,5%)	3 (17,6%) 1 (5,9%) 9 (52,9%) 4 (23,5%)	5 (12,5%) 3 (7,5%) 26 (65,0%) 6 (15,0%)
**Age of onset of main drug use** ^a^	21,0 ± 9,00	23,41 ± 8,76	19,98 ± 9,01
**Years of evolution since diagnosis** ^a^	18,38 ± 11,81	17,06 ± 10,76	18,94 ± 12,32
**Personality disorders** (Yes) ^b^	7 (12,3%)	1 (5,9%)	6 (15,0%)
**HIV** ^b^ • Negative • Positive • Unknown	52 (91,2%) 4 (7,0%) 1 (1,8%)	15 (88,2%) 2 (11,8%) 0 (0,0%)	37 (92,5%) 2 (5,0%) 1 (2,5%)
**Criminal conduct (Yes)** ^b^	10 (17,5%)	4 (23,5%)	6 (15,0%)
**Care modality** ^b^ • Outpatient clinic • Semi-residencial • Residencial	20 (35,1%) 8 (10,0%) 29 (50,9%)	8 (47,1%) 0 (0,0%) 9 (52,9%)	12 (30,0%) 8 (20,0%) 20 (50,0%)

SCI, Social Cognition Impairment; NSCI, No Social Cognition Impairment. a Mean ± SD; b n (%). Comparisons: Student’s t-test (continuous) or χ² test (categorical).

The sample of patients with SC impairment consisted of 40 individuals with a mean age of 47.68 years, of whom 31 (77.5%) were men and 9 (22.5%) were women. The mean duration of diagnosis was 18.94 years.

Regarding the initial SC screening, in the group of women with SC impairment, statistically significant differences were found for the emotions of fear (*p* = 0.007), disgust (*p* = 0.030), and the total EFT-60 score (*p* = 0.006), as well as for the empathic concern dimension (*p* = 0.036) of the IRI, compared to women without SC impairment. In the group of males with SC impairment, statistically significant differences were observed for the emotions of fear (*p* = 0.030), sadness (p = 0.002), disgust (*p* = .002), and the total EFT score (*p* = .001), as well as, for the fantasy dimension (*p* = 0.041) of the IRI, compared to male without SC impairment. **Table 3** shows the results of SC screening tests for all patients recruited in this study.

**Table 3:** Results of social cognition screening (EFT and IRI) in patients with and without Social Cognition Impairment (SCI), stratified by sex.

Social Cognition Test	Group of females				Group of males			
	N=18 (31.6%)				N=39 (68.4%)			
	NSCI group	SCI group	Statistics		NSCI group	SCI group	Statistics	
	N=9	N=9	U	*p*	N=9	N=9	U	*p*
EFT ^a^								
Joy	9.67 ± 0,50	9.56 ± 0,72	39.000	0.873	9.63 ± 0.52	9.39 ± 1.54	118.000	0.797
Surprise	9.33 ± 1.00	8.33 ± 1.50	22.000	0.087	9.38 ± 0.74	7.87 ± 2.58	81.500	0.125
Fear	6.22 ± 1.78	3.56 ± 2.18	10.500	0.007**	5.75 ± 2.86	3.32 ± 2.04	62.500	0.030*
Sadness	7.00 ± 1.50	6.22 ± 2.38	31.500	0.419	8.00 ± 1.51	5.52 ± 2.06	36.500	0.002**
Disgust	8.11 ± 1.27	6.11 ± 2.26	16.500	0.030*	8.25 ± 1.58	4.74 ± 2.85	34.000	0.002**
Anger	6.22 ± 2.72	5.89 ± 2.37	35.000	0.623	6.63 ± 1.20	5.68 ± 2.26	91.500	0.251
Total	48.33 ± 5.57	40.00 ± 6.53	9.500	0.006**	40.63 ± 4.93	37.29 ± 7.79	27.000	0.001**
IRI ^a^								
Perspective taking	9.56 ± 0.72	2.94 ± 0.95	35.500	0.657	2.95 ± 0.60	2.73 ± 0.56	98.000	0.363
Fancy	8.33 ± 1.50	1,94 ± 1.49	24.500	0.156	2.44 ± 0.57	1.78 ± 0.90	65.500	0.041*
Empathetic concern	3.56 ± 2.18	2.09 ± 1.64	17.000	0.036*	3.15 ± 0.33	2.62 ± 0.90	82.000	0.142
Personal anguish	6.22 ± 2.38	2.70 ± 1.07	18.500	0.051	1.60 ± 0.69	2.04 ± 1.06	97.500	0.355

SCI, Social Cognition Impairment; NSCI: without social cognition impairment, EFT, facial emotion recognition; IRI, empathy. Data: mean ± SD. Student’s t-test was used to analyze potential significant differences between mean scores measured in z-scores by sex. For group comparisons, Mann-Whitney U tests and independent samples t-tests were used according to variable distribution, with a significance level set at 5% (p < 0.05). Comparisons between clinical sample scores and normative values from the non-clinical population were conducted using one-sample t-tests. Significance levels: * *p* ≤.05; ** *p* ≤.01; *** *p* ≤.001.

Compared to normative values in the non-clinical population, the group of patients with SC impairment showed statistically significant differences for the emotions of happiness (*p* = 0.05), fear (*p* < 0.001), sadness (*p* < 0.001), disgust (*p* < 0.001), anger (*p* < 0.001), and the total EFT score (*p* < 0.001). Differences were also significant for the fantasy (*p* = 0.002) and personal distress (*p* = 0.042) dimensions of the IRI, as well as for the hostility (*p* < 0.001), intentionality (*p* < 0.001), and aggressiveness (*p* < 0.001) biases of the AIHQ. No significant differences were found in the ToM assessment using the Hinting Task. **Table 4** presents the analysis of scores for the group of patients with SC impairment compared to normative values in the non-clinical population.

**Table 4:** Comparison of scores in the social cognition impairment group with normative values from the non-clinical population.

Social cognition subdomains	Test	Normative values for the non-clinical population	SCI Group N=40	t	*p*
**EFT ^a^**					
Emotional Processing	Joy	9.87 ± 0.42	9.43 (± 1.39)	-2.090	0.050*
	Surprise	8.55 ± 1.44	7.98 (± 2.37)	-1.535	0.133
	Fear	7.19 ± 2.03	3.38 (± 2.05)	-11.789	<0.001***
	Sadness	8.33 ± 1.66	5.68 (± 2.13)	-7.887	<0.001***
	Disgust	8.59 ± 1.62	5.05 (± 2.76)	-8.101	<0.001***
	Anger	7.86 ± 1.90	5.73 (± 2.25)	-5.993	<0.001***
	Total	50.64 ± 5.04	37.90 (± 7.54)	-10.690	<0,001***
**IRI ^a^**					
Empathy	Perspective taken	2.69 ± 0.57	2.78 (± 0.66)	0.876	0.386
	Fancy	2.37 ± 0.84	1.83 (± 1.05)	-3.270	0.002**
	Empathetic concern	2.81 ± 0.64	2.50 (± 1.11)	-1.739	0.090
	Personal anguish	1.83 ± 0.69	2.19 (± 1.09)	2.101	0.042**
**AIHQ ^a^**					
Attributional style	Hostility	17.50 ± 8.69	32.10 (± 6.64)	13.898	<0.001***
	Intentionality	40.83 ± 7.43	47.17 (± 8.37)	4.793	<0.001***
	Anger	39.13 ± 8.12	39.85 (± 8.73)	0.522	0.605
	Guilt	38.63 ± 6.52	41.10 (± 9.33))	1.674	0.102
	Aggressiveness	21.70 ± 8.36	28.20 (7.57)	5.431	<0.001***
Theory of mind	**HINTING TASK ^a^**	18.31 ± 1.90	17.60 (± 2.33)	-1.928	0.06

SCI, Social Cognition Impairment. Data: mean ± SD. Comparisons were performed using one-sample t-tests (*p≤.05, **p≤.01, ***p≤.001). This comparison was conducted to assess the degree of clinical deviation of the SCI group relative to the expected performance in adults without psychiatric diagnoses or substance use history, based on widely used normative values in the literature. Sources of Normative Values: EFT (Ekman 60 Faces Test): Normative values from Young et al. (37); IRI (Interpersonal Reactivity Index): Reference values from the validated Portuguese adaptation by Limpo et al. (39); AIHQ (Ambiguous Intentions Hostility Questionnaire): Mean and standard deviation according to Combs et al. (41); Hinting Task: Normative values for healthy adults from Gil et al. (42).

The analysis of the results in the SC domains assessed in this study, in the SCI group, reveals slight differences between men and women, which do not reach statistical significance:

Emotional recognition (EFT): Male scored lower than female in the recognition of happiness (M=-1.14; W=-0.73), surprise (M=-0.47; W=-0.15), fear (M=-1.90; W=-1.66), sadness (M=-1.69; W=-1.27), disgust (M=-2.37; W=-1.53), anger (M=-1.14; W=-1.03), and in the total score (M=-2.78; W=-1.87).

Empathy dimensions (IRI): Females scored lower in empathic concern (M=-0.29; W=-1.12) and higher in personal distress (M=+0.30; W=+1.26).

Attributional style biases (AIHQ): Females scored higher than males in hostility bias (M=+1.70; W=+1.57) and aggressiveness bias (M=+0.81; W=+0.66), while males scored higher in intentionality bias (M=+0.78; W=+1.08), anger bias (M=-0.07; W=+0.65), and blame bias (M=+0.26; W=+0.77).

Theory of Mind (Hinting Task): Males showed slightly lower performance than females (M=-0.46; W=-0.04).

**Table 5** details the differences observed between males and females in the SC subdomains explored.

**Table 5:** Comparison between males and females with social cognition impairment across assessed subdomains.

Social cognition subdomains	Test	Females N=9	z	Males N=31	z	*p*
**EFT ^a^**						
Emotional Processing	Joy	9.56 ± 0.72	-0.73	9.39 ± 1.54	-1.14	0.841
	Surprise	8.33 ± 1.50	-0.15	7,87 ± 2.58	-0.47	0.921
	Fear	3.56 ± 2.18	-1.66	3,32 ± 2.04	-1.90	0.680
	Sadness	6,22 ± 2.38	-1.27	5,52 ± 2.06	-1.69	0.459
	Disgust	6,11 ± 2.26	-1.53	4,74 ± 2.85	-2.37	0.176
	Anger	5.89 ± 2.37	-1.03	5,68 ± 2.26	-1.14	0.818
	Total	40.00 ± 6.53	-1.87	37.29 ± 7.79	-2.78	0.592
**IRI ^a^**						
Empathy	Perspective taken	2.94 ± 0.95	+0.43	2.73 ± 0.56	+0.07	0.625
	Fancy	1.94 ± 1.49	-0.51	1.78 ± 0.90	-0.70	0.948
	Empathetic concern	2.09 ± 1.64	-1.12	2.62 ± 0.90	-0.29	0.660
	Personal anguish	2.70 ± 1.07	+1.26	2.04 ± 1.06	+0.30	0.130
**AIHQ ^a^**						
Attributional style	Hostility	31.22 ± 8.5	+1.57	32.35 ± 6.15	+1.70	0.897
	Intentionality	48.89 ± 8.95	+1.08	46.68 ± 8.28	+0.78	0.277
	Anger	44.44 ± 9.53	+0.65	38.52 ± 8.16	-0.07	0.077
	Guilt	43.67 ± 10.85	+0.77	40.35 ± 8.9	+0.26	0.224
	Aggressiveness	27.22 ± 5.49	+0.66	28.48 ± 8.13	+0.81	0.858
Theory of mind	**HINTING TASK ^a^**	18.22 ± 1.64	-0.04	17.42 ± 2.49	-0.46	0.296

EFT, emotion recognition; IRI, empathy; AIHQ, hostility bias. Data: ^a^ mean ± SD; z=Mann-Whitney U. * *p* ≤ 0.05; ** *p* ≤ 0.01; *** *p* ≤ 0.001; *** *p* ≤ 0.001.

## Discussion

This study aimed to examine the presence of SC dysfunctions in a clinical sample of individuals with SUD by evaluating the subdomains most referenced in the literature—emotional recognition, empathy, theory of mind, and attributional style—and to assess potential differences between males and females.

To our knowledge, this study represents the first work conducted with a clinical sample of the Portuguese population with SUD that reports results on the evaluation of emotional recognition, empathy, attributional biases, theory of mind, comparisons with clinical and non-clinical populations, and differences between males and females.

Given that this study was carried out in a clinical care setting, we considered it appropriate to conduct an initial screening of the recruited population to exclude patients who did not show at least minimal SC impairments. For this purpose, we chose to assess emotional recognition, the most studied subdomain in clinical samples (43), and empathy, a multidimensional construct referring to the ability to share and understand others’ subjective experiences. Empathy includes aspects of emotional communication, self-awareness, and theory of mind (32, 44). Using the criteria of scoring below 42 in emotional recognition, as measured by the EFT, and/or scoring more than one standard deviation below the mean in cognitive and/or emotional empathy, as measured by the IRI, we found that 70.2% of the recruited patients exhibited some SC impairment.

This result aligns with clinical experience and existing knowledge about how SC impairments contribute to the frequent social dysfunctions observed in the daily lives of individuals with SUD. Such impairments also play a role in therapeutic relationships, potentially hindering treatment success (45). These findings support the importance of addressing SC deficits during the care process for these patients (46). It has been proposed that SC deficits may represent a central cognitive phenotype for many developmental, neurological, and psychiatric disorders, with potential utility as clinical markers and a need for effective transdiagnostic interventions (47).

In our study, SC impairments were more frequent among males (79.5%) than females (50%), consistent with other studies indicating better performance by women in emotional recognition and empathy, as discussed below.

One of the most studied subdomains of SC is emotional recognition (48). Numerous studies have reported emotional recognition deficits in individuals with SUD, and a meta-analysis has confirmed impaired emotional processing in these patients, particularly in facial emotion recognition (43). While it has been suggested that deficits in facial emotion recognition are associated with the quantity, duration, and severity of polysubstance use (31), other evidence points to these deficits as potential predisposing factors for developing SUD (49, 50).

Additionally, poor emotional recognition performance at the start of treatment has been identified as a significant predictor of relapse/dropout (51). For instance, recently detoxified alcohol-dependent patients have shown deficits in emotional recognition linked to a higher frequency of interpersonal conflicts (52). These types of deficits undermine not only the ability to navigate daily social interactions but also the capacity to successfully engage in treatment programs.

In our study, we observed that patients with SUD exhibited significant impairment in emotion recognition, specifically in identifying fear, sadness, disgust, anger, and happiness. Furthermore, differences in the total scores of the facial emotion recognition (EFT score) task indicate that these individuals fail to accurately recognize basic emotions through facial expressions. When comparing SUD patients with non-clinical populations, we aimed to highlight the association between the severity of drug use and alterations in specific cognitive domains, such as social cognition.

This study provides a descriptive analysis of the current condition of these patients, focusing on the differences between SDI and NSDI as well as SDI and healthy controls. These findings align with research from Monash University (Australia), which demonstrated significantly worse facial emotion recognition, particularly for anger, disgust, fear, and sadness, in polysubstance users compared to healthy controls (31). However, they differ from others that did not identify impaired recognition of facial expressions (27). Such discrepancies may be attributed to conceptual factors, methodological diversity, and ecological validity issues in the tools used (53). The available evidence does not yet allow for establishing specific indications for specific emotions, but it provides compelling arguments for further study into the clinical utility of systematically assessing emotional processing in patients with SUD and implementing specific therapies that could be more relevant in the case of men and for emotions such as fear, disgust, and sadness.

Regarding potential gender differences, we found that males performed slightly worse than females in recognizing joy, surprise, fear, sadness, disgust, anger, and in the total EFT score, consistent with previous studies (54–56). Additionally, females with SC impairment showed significant differences in the recognition of fear, disgust, and total EFT scores compared to females without SC impairment. Among males, these differences were observed for fear, sadness, disgust, and total EFT scores. These findings support the relevance of addressing emotional processing deficits as a potential strategy to improve SUD treatment outcomes (34).

When discussing the ToM, it refers to the ability to attribute mental states to oneself and others (57–59). This hetero metacognitive skill enables understanding and predicting others’ behavior, perceptions, knowledge, beliefs, goals, and intentions (60). ToM encompasses cognitive ToM (inferences about thoughts) and affective ToM (inferences about feelings). Various tests assess this complex construct, evaluating tasks such as understanding false beliefs, deception, white lies, jokes, metaphors, irony, hints, and faux pas (61). One of the most referenced tools in the literature is the hinting task, which examines the ability to infer intentions behind indirect speech (40, 42).

Numerous studies have reported ToM impairments across various conditions, including neurological and mental disorders such as autism spectrum disorders, schizophrenia, borderline personality disorder, post-traumatic stress disorder, depression, and eating disorders (62), as well as SUD (32, 63).

In our study, results from the hinting task were similar to those of non-clinical populations, indicating average performance. Previous research has identified ToM impairments in individuals with SUD using tools like the Movie for the Assessment of Social Cognition (MASC) (64) or the Reading the Mind in the Eyes Test (RMET) (65). A meta-analysis supports that drug users perform worse than healthy controls, with stronger evidence for ToM impairments in studies on alcohol and methamphetamine dependence, mixed findings for cocaine users, and no ToM impairments in recreational cannabis or cocaine users. However, the analysis highlights various biases that hinder generalizing these findings (32).

In terms of gender differences, males in our study performed slightly worse than females on the hinting task, consistent with a recent meta-analysis confirming better ToM performance in females (66).

Empathy, a multidimensional construct, can be defined as the processes and outcomes related to an individual’s responses to another’s experiences, encompassing cognitive and affective aspects (67, 68). Empathy involves the ability to adopt others’ perspectives, infer mental states, and share their cognitive and emotional experiences. It includes cognitive empathy (perspective-taking and fantasy) and affective empathy (empathic concern and personal distress) (38, 69). Brain injuries and psychiatric disorders such as antisocial, borderline, and narcissistic personality disorders, autism spectrum disorders, schizophrenia, and alexithymia are associated with empathy deficits or absence (44, 70). Evidence also points to empathy impairments in SUD patients and suggests that improving empathic behaviors can enhance treatment outcomes and reduce relapse risk (71, 72).

In our study, the IRI revealed slightly different results compared to non-clinical populations, with lower fantasy and higher personal distress in the SC impaired group, without significant gender differences across the four dimensions assessed. However, SC impaired females showed lower empathic concern than their unimpaired counterparts, while SC impaired males showed differences in the fantasy dimension. These findings align with reports of reduced empathic processes in individuals with SUD, including alcohol, opioid, cocaine, methamphetamine, and polysubstance users (34, 72, 73). While it is widely accepted that females exhibit higher empathy levels than males (72, 74), gender differences in clinical populations remain underexplored, and findings are contradictory (71, 75).

Attributional style refers to how individuals explain the causes of events or social interactions (76). Several models and theories have been proposed to explain attributional processes and their role in the pathogenesis and clinical features of mental disorders (77–80). Dimensions such as internality/externality, stability/instability, globality/specificity, consensus, distinctiveness, and consistency have been identified, and certain attributional biases have been linked to various clinical conditions. Although attributional style is understudied in SUD populations, evidence suggests that neuropsychological impairments and inappropriate explanatory styles are prognostic factors in treatment. Tailoring therapeutic interventions to recover these impaired functions and implementing specific rehabilitation strategies could enhance motivation, adherence, and reduce relapse risk (81).

The hostile attribution bias, defined as the tendency to interpret others’ behavior as having hostile intent, particularly in ambiguous social contexts (82), has been associated with interpersonal conflicts, paranoia, anxiety, mood disorders, and schizophrenia spectrum disorders (83). It has also been studied in severe alcohol use disorder patients (84). In our study, SC impaired patients scored higher in hostility, intentionality, and aggressiveness dimensions of the AIHQ compared to non-clinical norms. Regarding gender differences, males showed slightly higher hostility and aggressiveness scores, while females scored higher in intentionality, anger, and guilt.

Overall, the results of this study highlight the clinical relevance of SC impairments in SUD patients, given their frequency, complex implications for pathogenesis, progression, and therapeutic response, and their potential as a viable target for improving care processes. Neuroscience has provided deeper insights into the underlying causes of behavioral patterns in addiction disorders, emphasizing the role of brain mechanisms related to reward systems, learning, motivation, cognition, and executive functioning (85–88). From this perspective, SC emerges as another functional domain of interest for further research into SUD and its potential utility in enhancing therapeutic strategies.

### Study limitations

Because our study focuses on characterizing social cognition functionality specifically in patients with SUD who present social cognition impairment (SCI), the findings cannot be generalized to all SUD patients except regarding: 1) The prevalence rate of SCI in this clinical sample and 2) Observed gender differences in SCI presentation.

This study has several limitations that should be considered. First, the relatively small sample size may restrict the statistical power and generalizability of the findings, potentially masking subtle effects or interactions. Second, the underrepresentation of women in the sample limits the ability to explore potential gender differences in social cognition, which have been reported in prior addiction research. Finally, the reliance on specific behavioral or self-report measures of social cognition (e.g., theory of mind or emotion recognition tasks) may not fully capture the complexity of real-world social functioning.

A key limitation stems from how the SCI group was operationally defined through specific cutoff scores on screening instruments (EFT and IRI). This approach identifies a pre-screened subgroup within the overall clinical sample and does not represent the full clinical diversity of SUD populations.

Limits external validity when extrapolating beyond this subgroup. The SCI group composition reflected operational criteria designed to detect SC deficits, which may overlook dimensional variations across social cognition domains and require validation through dimensional analyses across the full SUD sample and inclusion of matched control groups in future studies. Future studies with larger, more balanced cohorts and multimodal assessments (e.g., ecological paradigms, neuroimaging) could help address these constraints.

## Conclusions

This study has replicated findings regarding impairments in SC subdomains related to emotional recognition, empathy, and attributional style in a heterogeneous clinical sample of individuals with SUD. These deficits have high clinical relevance, as they have been consistently linked to interpersonal problems, increased social stress, higher rates of treatment dropout, and relapses.

In the future, it would be crucial to consider the systematic evaluation of SC in diagnostic protocols for individuals with SUD, which would better identify the difficulties that interfere with the social functionality of these patients, help reduce early treatment dropout, and prevent relapses. Additionally, the clinical utility of SC is emphasized as an important prognostic variable to improve the personalization of the therapeutic process and rehabilitation.

## Data Availability

The original contributions presented in the study are included in the article/supplementary material. Further inquiries can be directed to the corresponding author.
